# Supplementary material for: Introducing riskCommunicator: An R package to obtain interpretable effect estimates for public health
Source: PLoS One. 2022 Jul 18;17(7):e0265368. doi: 10.1371/journal.pone.0265368 (PMC9292119; doi:10.1371/journal.pone.0265368)
Supplement: S1 Appendix — (PDF) [file pone.0265368.s001.pdf]

riskCommunicator Manuscript Vignette

Jessica Grembi, Elizabeth Rogawski McQuade

2022-05-26

Contents

Introduction to `riskCommunicator` 1

Getting started 1

Installation . . . 1

Description of main package function . . . 2

Framingham Heart Study 5

Binary outcome example . . . 5

Rate outcome example . . . 10

Rate outcome with subgroups example . . . 11

Introduction to `riskCommunicator`

The `riskCommunicator` package facilitates the estimation of common epidemiological effect measures that are relevant to public health, but that are often not trivial to obtain from common regression models, like logistic regression. In particular, `riskCommunicator` estimates risk and rate differences, in addition to risk and rate ratios. The package estimates these effects using g-computation with the appropriate parametric model depending on the outcome (logistic regression for binary outcomes, Poisson regression for rate or count outcomes, negative binomial regression for overdispersed rate or count outcomes, and linear regression for continuous outcomes). Therefore, the package can handle binary, rate, count, and continuous outcomes and allows for dichotomous, categorical (>2 categories), or continuous exposure variables. Additional features include estimation of effects stratified by subgroup and adjustment of standard errors for clustering. Confidence intervals are constructed by bootstrap at the individual or cluster level, as appropriate.

This package operationalizes g-computation, which has not been widely adopted due to computational complexity, in an easy-to-use implementation tool to increase the reporting of more interpretable epidemiological results. To make the package accessible to a broad range of health researchers, our goal was to design a function that was as straightforward as the standard logistic regression functions in R (e.g. `glm`) and that would require little to no expertise in causal inference methods or advanced coding.

Getting started

Installation

The `riskCommunicator` R package is available from CRAN so can be installed using the following command:

```r
install.packages("riskCommunicator")
```

Load packages:

```r
library(riskCommunicator)
library(tidyverse)
#> -- Attaching packages ------------------- tidyverse 1.3.1 --
#> v ggplot2 3.3.6     v purrr   0.3.4
#> v tibble  3.1.7     v dplyr   1.0.9
#> v tidyr   1.2.0     v stringr 1.4.0
#> v readr   2.1.2     v forcats 0.5.1
#> -- Conflicts ---------------------- tidyverse_conflicts() --
#> x dplyr::filter() masks stats::filter()
#> x dplyr::lag()    masks stats::lag()
library(printr)
#> Registered S3 method overwritten by 'printr':
#>   method                from
#>   knit_print.data.frame rmarkdown
library(formatR)
library(sandwich)
library(stringr)
library(ggpubr)
```

Description of main package function

The `gComp` function is the main function in the `riskCommunicator` package and allows you to estimate a variety of effects depending on your outcome and exposure of interest. The function is coded as follows:

```r
?gComp
#> Estimate difference and ratio effects with 95% confidence intervals.
#>
#> Usage:
#>
#>      gComp(
#>        data,
#>        outcome.type = c("binary", "count", "count_nb", "rate", "rate_nb", "continuous"),
#>        formula = NULL,
#>        Y = NULL,
#>        X = NULL,
#>        Z = NULL,
#>        subgroup = NULL,
#>        offset = NULL,
#>        rate.multiplier = 1,
#>        exposure.scalar = 1,
#>        R = 200,
#>        clusterID = NULL,
#>        parallel = "no",
#>        ncpus = getOption("boot.ncpus", 1L)
#>      )
#>
#> Arguments:
#>
```

```
#>     data: (Required) A data.frame containing variables for 'Y', 'X',
#>           and 'Z' or with variables matching the model variables
#>           specified in a user-supplied formula. Data set should also
#>           contain variables for the optional 'subgroup' and 'offset',
#>           if they are specified.
#>
#> outcome.type: (Required) Character argument to describe the outcome
#>           type. Acceptable responses, and the corresponding error
#>           distribution and link function used in the 'glm', include:
#>
#>           binary (Default) A binomial distribution with link = 'logit'
#>               is used.
#>
#>           count A Poisson distribution with link = 'log' is used.
#>
#>           count_nb A negative binomial model with link = 'log' is used,
#>               where the theta parameter is estimated internally; ideal
#>               for over-dispersed count data.
#>
#>           rate A Poisson distribution with link = 'log' is used; ideal
#>               for events/person-time outcomes.
#>
#>           rate_nb A negative binomial model with link = 'log' is used,
#>               where the theta parameter is estimated internally; ideal
#>               for over-dispersed events/person-time outcomes.
#>
#>           continuous A gaussian distribution with link = 'identity' is
#>               used.
#>
#>  formula: (Optional) Default NULL. An object of class "formula" (or one
#>           that can be coerced to that class) which provides the the
#>           complete model formula, similar to the formula for the glm
#>           function in R (e.g. `Y ~ X + Z1 + Z2 + Z3`). Can be supplied
#>           as a character or formula object. If no formula is provided,
#>           Y and X must be provided.
#>
#>        Y: (Optional) Default NULL. Character argument which specifies
#>           the outcome variable. Can optionally provide a formula
#>           instead of 'Y' and 'X' variables.
#>
#>        X: (Optional) Default NULL. Character argument which specifies
#>           the exposure variable (or treatment group assignment), which
#>           can be binary, categorical, or continuous. This variable can
#>           be supplied as a factor variable (for binary or categorical
#>           exposures) or a continuous variable. For binary/categorical
#>           exposures, 'X' should be supplied as a factor with the lowest
#>           level set to the desired referent. Numeric variables are
#>           accepted, but will be centered (see Note). Character
#>           variables are not accepted and will throw an error. Can
#>           optionally provide a formula instead of 'Y' and 'X'
#>           variables.
#>
#>        Z: (Optional) Default NULL. List or single character vector
```

```
#>           which specifies the names of covariates or other variables to
#>           adjust for in the 'glm' function. All variables should either
#>           be factors, continuous, or coded 0/1 (i.e. not character
#>           variables). Does not allow interaction terms.
#>
#> subgroup: (Optional) Default NULL. Character argument that indicates
#>           subgroups for stratified analysis. Effects will be reported
#>           for each category of the subgroup variable. Variable will be
#>           automatically converted to a factor if not already.
#>
#>   offset: (Optional, only applicable for rate/count outcomes) Default
#>           NULL. Character argument which specifies the variable name to
#>           be used as the person-time denominator for rate outcomes to
#>           be included as an offset in the Poisson regression model.
#>           Numeric variable should be on the linear scale; function will
#>           take natural log before including in the model.
#>
#> rate.multiplier: (Optional, only applicable for rate/count outcomes).
#>           Default 1. Numeric variable signifying the person-time value
#>           to use in predictions; the offset variable will be set to
#>           this when predicting under the counterfactual conditions.
#>           This value should be set to the person-time denominator
#>           desired for the rate difference measure and must be inputted
#>           in the units of the original offset variable (e.g. if the
#>           offset variable is in days and the desired rate difference is
#>           the rate per 100 person-years, rate.multiplier should be
#>           inputted as 365.25*100).
#>
#> exposure.scalar: (Optional, only applicable for continuous exposure)
#>           Default 1. Numeric value to scale effects with a continuous
#>           exposure. This option facilitates reporting effects for an
#>           interpretable contrast (i.e.  magnitude of difference) within
#>           the continuous exposure. For example, if the continuous
#>           exposure is age in years, a multiplier of 10 would result in
#>           estimates per 10-year increase in age rather than per a
#>           1-year increase in age.
#>
#>        R: (Optional) Default 200. The number of data resamples to be
#>           conducted to produce the bootstrap confidence interval of the
#>           estimate.
#>
#> clusterID: (Optional) Default NULL. Character argument which specifies
#>           the variable name for the unique identifier for clusters.
#>           This option specifies that clustering should be accounted for
#>           in the calculation of confidence intervals. The 'clusterID'
#>           will be used as the level for resampling in the bootstrap
#>           procedure.
#>
#> parallel: (Optional) Default "no." The type of parallel operation to be
#>           used. Available options (besides the default of no parallel
#>           processing) include "multicore" (not available for Windows)
#>           or "snow." This argument is passed directly to 'boot'. See
#>           note below about setting seeds and parallel computing.
```

```
#>
#>    ncpus: (Optional, only used if parallel is set to "multicore" or
#>           "snow") Default 1.  Integer argument for the number of CPUs
#>           available for parallel processing/ number of parallel
#>           operations to be used.  This argument is passed directly to
#>           'boot'
```

Framingham Heart Study

We'll demonstrate how to use the package with data from the Framingham Heart Study. The following information is from the official Framingham study documentation (https://biolincc.nhlbi.nih.gov/teaching/):

"The Framingham Heart Study is a long term prospective study of the etiology of cardiovascular disease among a population of free living subjects in the community of Framingham, Massachusetts. The Framingham Heart Study was a landmark study in epidemiology in that it was the first prospective study of cardiovascular disease and identified the concept of risk factors and their joint effects. The study began in 1948 and 5,209 subjects were initially enrolled in the study. Participants have been examined biennially since the inception of the study and all subjects are continuously followed through regular surveillance for cardiovascular outcomes. Clinic examination data has included cardiovascular disease risk factors and markers of disease such as blood pressure, blood chemistry, lung function, smoking history, health behaviors, ECG tracings, Echocardiography, and medication use. Through regular surveillance of area hospitals, participant contact, and death certificates, the Framingham Heart Study reviews and adjudicates events for the occurrence of Angina Pectoris, Myocardial Infarction, Heart Failure, and Cerebrovascular disease.

```
data(cvdd)
```

cvdd is a subset of the data collected as part of the Framingham study from 4,240 participants who conducted a baseline exam and were free of prevalent coronary heart disease when they entered the study. Participant clinic data was collected during three examination periods, approximately 6 years apart, from roughly 1956 to 1968. Each participant was followed for a total of 24 years for the outcome of the following events: Angina Pectoris, Myocardial Infarction, Atherothrombotic Infarction or Cerebral Hemorrhage (Stroke) or death.

NOTE: This is a "teaching" dataset. Specific methods were employed to ensure an anonymous dataset that protects patient confidentiality; therefore, this dataset is inappropriate for publication purposes." The use of these data for the purposes of this package were approved on 11Mar2019 (request #7161) by NIH/NHLBI.

Binary outcome example

Research question: what is the effect of having diabetes at the beginning of the study on the 24-year risk of cardiovascular disease or death due to any cause?

Here, we will estimate the risk difference, risk ratio, odds ratio, and number needed to treat, adjusting for patient's age, sex, body mass index (BMI), smoking status (current smoker or not), and prevalence of hypertension (if they are hypertensive or not at baseline). Logistic regression is used as the underlying parametric model for g-computation.

```
## Specify the regression formula
cvdd.formula <- cvd_dth ~ DIABETES + AGE + SEX + BMI + CURSMOKE + PREVHYP

## For reproducibility, we should always set the seed since the g-computation uses
## random resampling of the data to calculate confidence intervals and random
## sampling of the distribution when predicting outcomes.
```

```r
set.seed(1298)

## Call the gComp function
binary.res <- gComp(data = cvdd,
                    formula = cvdd.formula,
                    outcome.type = "binary",
                    R = 1000)

binary.res
#> Formula:
#> cvd_dth ~ DIABETES + AGE + SEX + BMI + CURSMOKE + PREVHYP
#>
#> Parameter estimates:
#>                             DIABETES1_v._DIABETES0 Estimate (95% CI)
#> Risk Difference                                   0.287 (0.196, 0.395)
#> Risk Ratio                                        1.700 (1.476, 1.966)
#> Odds Ratio                                        4.550 (2.771, 9.085)
#> Number needed to treat/harm                                      3.484
```

The result obtained from the `gComp` function is an object of class **gComp** which is a list containing the summary results, `results.df`, `n`, `R`, `boot.result`, `contrast`, `family`, `formula`, `predicted.outcome`, and `glm.result` (see `?gComp` or `help(gComp)` for a more detailed explanation of each item in the list).

```r
class(binary.res)
#> [1] "gComp" "list"

## The names of the different items in the list:
names(binary.res)
#>  [1] "summary"           "results.df"
#>  [3] "n"                 "R"
#>  [5] "boot.result"       "contrast"
#>  [7] "family"            "formula"
#>  [9] "predicted.outcome" "glm.result"

## Sample size of the original data:
binary.res$n
#> [1] 4240

## Contrast being compared in the analysis:
binary.res$contrast
#> [1] "DIABETES1 v. DIABETES0"
```

There is also a summary method for objects with class **gComp** that contains the formula, family and link function, contrast being made, parameter estimates with 95% CIs, and a summary of the underlying glm used for predictions.

```r
summary(binary.res)
#> Formula:
#> cvd_dth ~ DIABETES + AGE + SEX + BMI + CURSMOKE + PREVHYP
#>
#> Family: binomial
#> Link function: logit
#>
```

```
#> Contrast: DIABETES1 v. DIABETES0
#>
#> Parameter estimates:
#>                              DIABETES1_v._DIABETES0 Estimate (95% CI)
#> Risk Difference                                    0.287 (0.196, 0.395)
#> Risk Ratio                                         1.700 (1.476, 1.966)
#> Odds Ratio                                         4.550 (2.771, 9.085)
#> Number needed to treat/harm                                       3.484
#>
#> Underlying glm:
#> Call:  stats::glm(formula = formula, family = family, data = working.df,
#>     na.action = stats::na.omit)
#>
#> Coefficients:
#> (Intercept)    DIABETES1          AGE         SEX1
#>    -6.25839      1.51501      0.10246     -0.79405
#>         BMI    CURSMOKE1     PREVHYP1
#>     0.02512      0.58550      0.77804
#>
#> Degrees of Freedom: 4220 Total (i.e. Null);  4214 Residual
#>   (19 observations deleted due to missingness)
#> Null Deviance:       5735
#> Residual Deviance: 4697  AIC: 4711
```

Checking model fit

The 95% CIs obtained from the riskCommunicator package represent population-standardized marginal effects obtained with g-computation. To ensure that the parameter estimates from each bootstrap iteration are normally distributed, we can also look at the histogram and Q-Q plots of bootstrapped estimates by calling:

```
plot(binary.res)
```

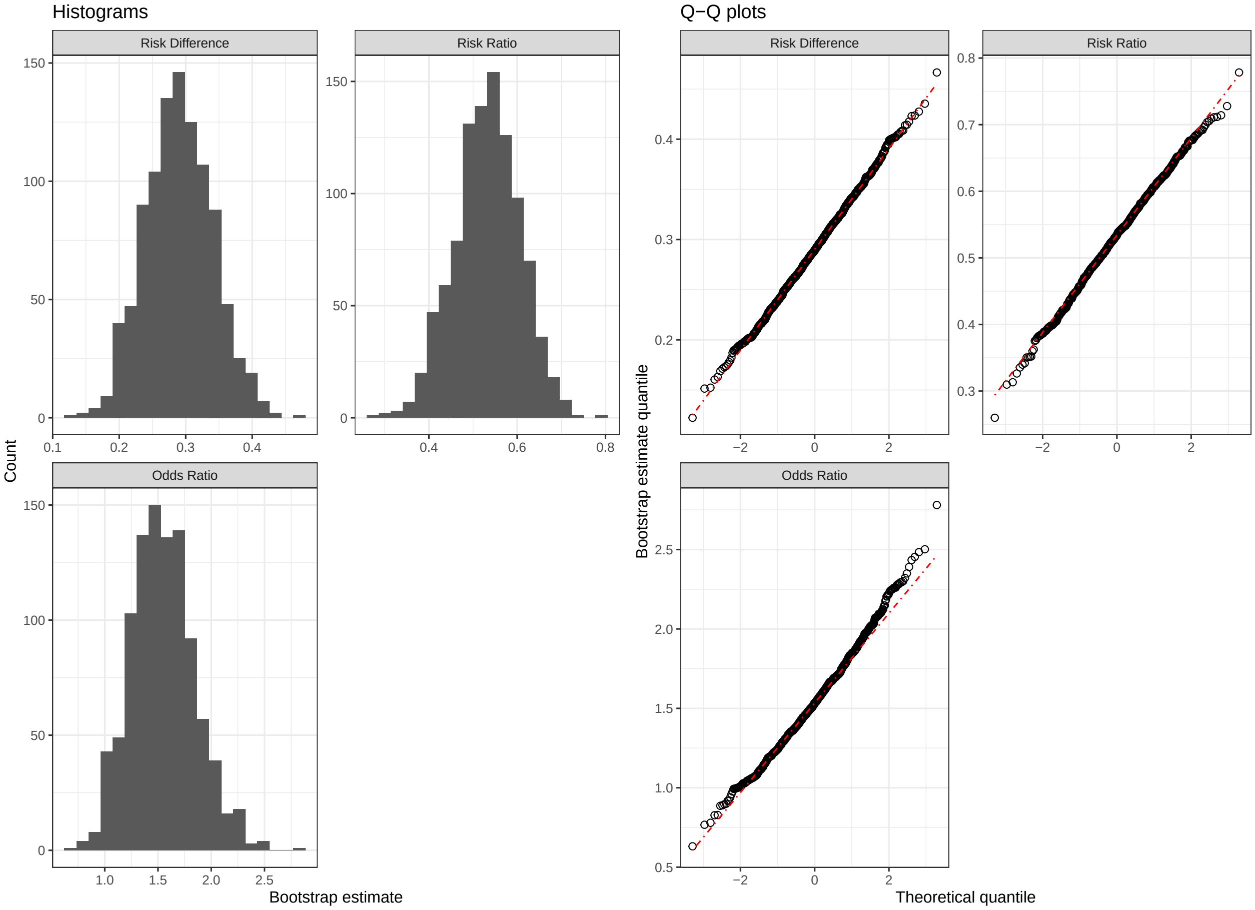

NOTE: All ratio values are plotted as natural log of the actual estimate

The histograms show the different effect estimates obtained by each bootstrap resampling of the data and should be normally distributed if the model is correctly specified. Q-Q plots help to verify that the bootstrap values are normally distributed by comparing the actual distribution of bootstrap values against a theoretical normal distribution of values centered at mean = 0. If the estimates are normally distributed, the plotted estimates (black circles) should overlay the diagonal red dashed line.

In the manuscript, we compare the results of gComp to standard regression models. Here, we show how to do that so we can re-create Table 2.

First we obtain the odds ratio using logistic regression.

```
#>
#> Call:  glm(formula = cvd_dth ~ DIABETES + AGE + SEX + BMI + CURSMOKE +
#>     PREVHYP, family = binomial(link = "logit"), data = cvdd)
#>
#> Coefficients:
#> (Intercept)    DIABETES1          AGE         SEX1
#>    -6.25839      1.51501      0.10246     -0.79405
#>         BMI    CURSMOKE1     PREVHYP1
#>     0.02512      0.58550      0.77804
#>
#> Degrees of Freedom: 4220 Total (i.e. Null);  4214 Residual
#>   (19 observations deleted due to missingness)
#> Null Deviance:       5735
```

```
#> Residual Deviance: 4697  AIC: 4711
```

Note the use of `confint.default` in the above call. The typical call `confint` does not return Wald-based CIs, so we've forced it with `confint.default`. You can read more about that here: https://stats.stackexchange.com/questions/5304/why-is-there-a-difference-between-manually-calculating-a-logistic-regression-95

Next, we calculate the risk ratio using a Poisson approximation of log-binomial regression with robust variance (sandwich standard errors).

```
#>
#> Call:  glm(formula = cvd_dth ~ DIABETES + AGE + SEX + BMI + CURSMOKE +
#>     PREVHYP, family = "poisson", data = cvdd %>% mutate(cvd_dth = ifelse(cvd_dth ==
#>     "0", 0, ifelse(cvd_dth == "1", 1, NA))))
#>
#> Coefficients:
#> (Intercept)    DIABETES1          AGE         SEX1
#>    -3.86603      0.39673      0.04930     -0.38348
#>         BMI    CURSMOKE1     PREVHYP1
#>     0.01403      0.26329      0.35452
#>
#> Degrees of Freedom: 4220 Total (i.e. Null);  4214 Residual
#>   (19 observations deleted due to missingness)
#> Null Deviance:       3079
#> Residual Deviance: 2539  AIC: 6073
```

We can try to calculate the risk difference using a log-linear regression, but the model won't converge.

```
std.reg.rd = glm(formula = cvd_dth ~ DIABETES + AGE + SEX + BMI + CURSMOKE + PREVHYP,
                 data = cvdd %>%
                   ## To use linear regression, we need to change DIABETES from a
                   ##   factor to a numeric (0,1) variable
                   mutate(cvd_dth = ifelse(cvd_dth == "0", 0,
                                          ifelse(cvd_dth == "1", 1, NA))),
                 family=gaussian(link = 'log'))
```

We can re-create Table 2 from the manuscript now!

```
# combine standard regression results
std.regression.res = df.std.reg.or %>%
  mutate(Parameter = "Odds Ratio",
         Std_regression = paste0(round(OR, 2),
                                 " (",
                                 round(LL, 2),
                                 ", ",
                                 round(UL, 2),
                                 ")")) %>%
  bind_rows(df.std.reg.rr %>%
              mutate(Parameter = "Risk Ratio",
                     Std_regression = paste0(round(Estimate, 2),
                                             " (",
                                             round(LL, 2),
                                             ", ",
                                             round(UL, 2),
```

```
                                             ")"))) %>%
  select(Parameter, Std_regression) %>%
  rename(`Standard regression` = Std_regression)
rownames(std.regression.res) = NULL


(table2 = binary.res$results.df %>%
  mutate(riskCommunicator = paste0(format(round(Estimate, 2), 2),
                                   " (",
                                   format(round(`2.5% CL`, 2), 2),
                                   ", ",
                                   format(round(`97.5% CL`, 2), 2),
                                   ")"),
         riskCommunicator = ifelse(Parameter == "Number needed to treat/harm",
                                   round(Estimate, 2), riskCommunicator)) %>%
  select(Parameter, riskCommunicator) %>%
  left_join(std.regression.res, by = "Parameter"))
```

Parameter	riskCommunicator	Standard regression
Risk Difference	0.29 (0.20, 0.39)	NA
Risk Ratio	1.70 (1.48, 1.97)	1.49 (1.33, 1.66)
Odds Ratio	4.55 (2.77, 9.09)	4.55 (2.66, 7.78)
Number needed to treat/harm	3.48	NA

Rate outcome example

Research question: what is the effect of having diabetes at the beginning of the study on the rate of cardiovascular disease or death due to any cause?

Here, we will estimate the rate difference and rate ratio, adjusting for patient's age, sex, body mass index (BMI), smoking status (current smoker or not), and prevalence of hypertension (if they are hypertensive or not at baseline). We have included **timeout** as the `offset` and a `rate.multiplier` of `365.25*100` so that the estimates are returned with units of 100 person-years. Poisson regression is used as the underlying parametric model for g-computation. (Note: for overdispersed count/rate outcomes, the negative binomial distribution can be specified by setting `outcome.type` to **"count_nb"** or **"rate_nb"**.)

```
## Modify the dataset to change the variable cvd_dth from a factor
##  to a numeric variable since the outcome for Poisson
##  regression must be numeric.
cvdd.t <- cvdd %>%
  dplyr::mutate(cvd_dth = as.numeric(as.character(cvd_dth)),
                timeout = as.numeric(timeout))

set.seed(6534)

rate.res <- gComp(data = cvdd.t,
                  Y = "cvd_dth",
                  X = "DIABETES",
                  Z = c("AGE", "SEX", "BMI", "CURSMOKE", "PREVHYP"),
                  outcome.type = "rate",
                  rate.multiplier = 365.25*100,
```

```
                  offset = "timeout",
                  R = 1000)

rate.res
#> Formula:
#> cvd_dth ~ DIABETES + AGE + SEX + BMI + CURSMOKE + PREVHYP + offset(log(timeout_adj))
#>
#> Parameter estimates:
#>                          DIABETES1_v._DIABETES0 Estimate (95% CI)
#> Incidence Rate Difference                    2.189 (1.436, 3.063)
#> Incidence Rate Ratio                         1.913 (1.603, 2.288)
```

Rate outcome with subgroups example

Research question: what is the effect of having diabetes at the beginning of the study on the rate of cardiovascular disease or death due to any cause, stratified by sex?

Here, we will estimate the same effects above, but in subgroups defined by sex.

```
rate.res.subgroup <- gComp(data = cvdd.t,
                           Y = "cvd_dth",
                           X = "DIABETES",
                           Z = c("AGE", "SEX", "BMI", "CURSMOKE", "PREVHYP"),
                           subgroup = "SEX",
                           outcome.type = "rate",
                           rate.multiplier = 365.25*100,
                           offset = "timeout",
                           R = 1000)

rate.res.subgroup
#> Formula:
#> cvd_dth ~ DIABETES + AGE + SEX + BMI + CURSMOKE + PREVHYP + DIABETES:SEX +      offset(log(timeout_a
#>
#> Parameter estimates:
#>                          DIABETES1_v._DIABETES0_SEX0 Estimate (95% CI)
#> Incidence Rate Difference                         2.488 (1.340, 4.058)
#> Incidence Rate Ratio                              1.794 (1.423, 2.301)
#>                          DIABETES1_v._DIABETES0_SEX1 Estimate (95% CI)
#> Incidence Rate Difference                         1.918 (0.995, 3.322)
#> Incidence Rate Ratio                              2.044 (1.541, 2.860)
```

The `results.df` component of the `gComp` function output is formatted as a data.frame. This makes it very easy to immediately plot the results using ggplot2 or your favorite R plotting functionality. Here's an example for plotting the results of the different subgroups (sexes) for the rate example above.

```
# Saving the output and modifying the labels of the SEX variable to specify
#   male/female instead of 0/1
# Also adding a new variable to show the line indicating no difference found
#   (0 for rate difference, 1 for rate ratio)
```

```
df = rate.res.subgroup$results.df %>%
  mutate(Subgroup = ifelse(Subgroup == "SEX0", "Male", "Female"),
         hline = ifelse(Parameter == "Incidence Rate Ratio", 1, 0))

ggplot(df, aes(x = Subgroup, y = Estimate)) +
  geom_point(size = 2) +
  geom_errorbar(aes(ymin = `2.5% CL`, ymax = `97.5% CL`),
                width = 0.2) +
  facet_wrap(~Parameter) +
  theme_bw() +
  labs(x = "", color = "") +
  geom_hline(aes(yintercept = hline),
             color = "red",
             linetype = "dashed",
             alpha = 0.3)
```

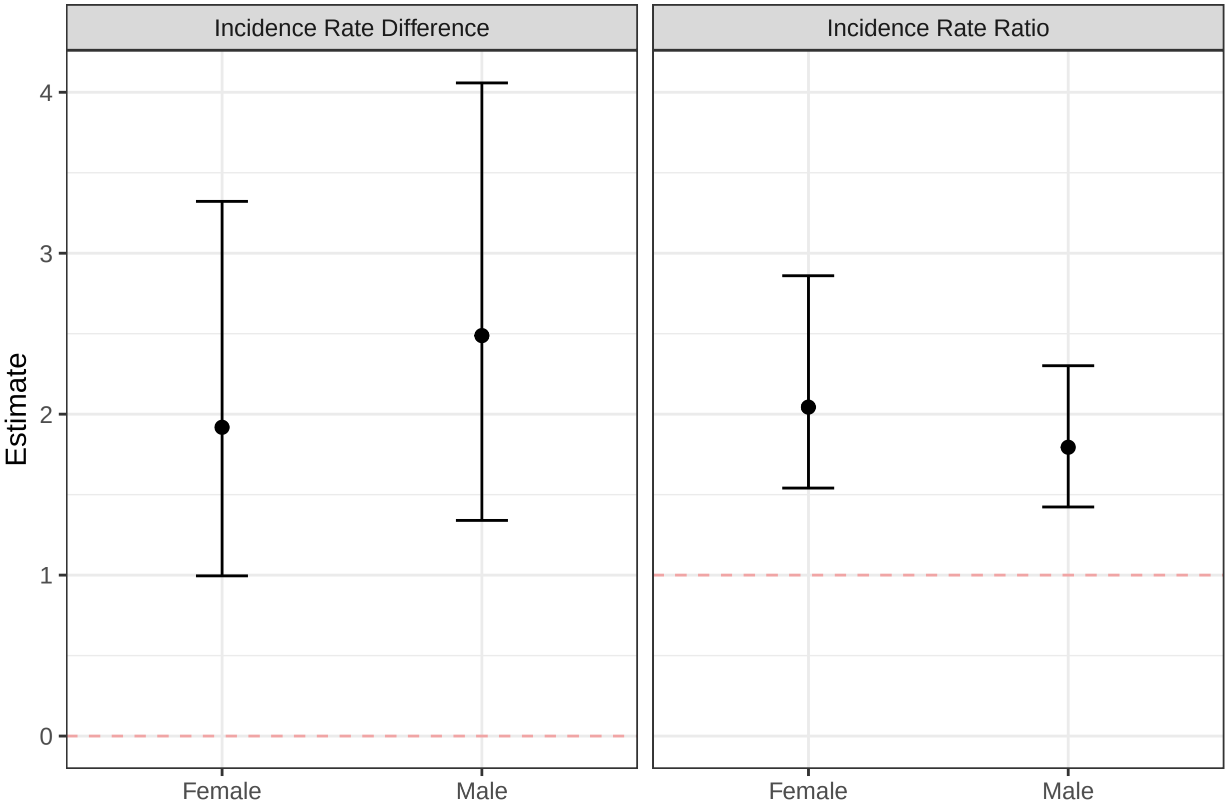

Let's make a figure comparing the results we got above using riskCommunicator with those we get using standard regression models for this rate example.

First, we need to get the covariate-conditional estimates using standard Poisson regression.

```
# Standard Poisson regression (spr) for the rate question, across both sexes
spr.rate = glm(
  formula = cvd_dth ~ DIABETES + AGE + SEX + BMI + CURSMOKE + PREVHYP +
    offset(log(timeout+0.001)),
  data = cvdd.t,
```

```
  family = "poisson"
  )

# get the parameter estimate and CI from the model object
df.spr.rate = as.data.frame(
  exp(cbind(Estimate = coef(spr.rate), confint.default(spr.rate, level = 0.95)))
  ) %>%
  rename(`2.5% CL` = `2.5 %`, `97.5% CL` = `97.5 %`) %>%
  filter(rownames(.) == "DIABETES1") %>%
  mutate(Subgroup = "All")


# Standard Poisson regression (spr) for the rate question, by subgroup (SEX)
spr.rate.subgroup = glm(
  formula = cvd_dth ~ DIABETES + AGE + SEX + BMI + CURSMOKE + PREVHYP +
    DIABETES*SEX + offset(log(timeout+0.001)),
  data = cvdd.t,
  family = "poisson"
  )

# Get the variance-covariance matrix so we can calculate standard errors
se.subgroup = vcov(spr.rate.subgroup)

# Get estimates and CIs
df.spr.rate.subgroup = data.frame(
  Subgroup = c("Male", "Female"),
  raw.est = c(coef(spr.rate.subgroup)[2],
              coef(spr.rate.subgroup)[2] + coef(spr.rate.subgroup)[8]),
  SE = c(sqrt(se.subgroup[2,2]),
         sqrt(se.subgroup[2,2] + se.subgroup[8,8] + 2*se.subgroup[2,8]))) %>%
  mutate(Estimate = exp(raw.est),
         `2.5% CL` = exp(raw.est - 1.96 * SE),
         `97.5% CL` = exp(raw.est + 1.96 * SE))

# Combine the results from the subgroup and full model into a single data.frame
combined.std.reg = df.spr.rate %>%
  bind_rows(df.spr.rate.subgroup %>%
            select(Subgroup, Estimate:`97.5% CL`)) %>%
  mutate(Parameter = "Incidence Rate Ratio",
         model = "Standard Poisson Regression")
```

Now, we can plot the same figure shown in the manuscript.

```
# Combine the riskCommunicator results with the standard Poisson regression results
df.combined = rate.res$results.df %>%
  mutate(Subgroup = "All") %>%
  bind_rows(df) %>%
  mutate(model = "riskCommunicator") %>%
  select(-Outcome, -Comparison) %>%
  bind_rows(combined.std.reg) %>%
  mutate(hline = ifelse(Parameter == "Incidence Rate Ratio", 1, 0))
```

```
rate.diff = ggplot(df.combined %>%
                     filter(Parameter == "Incidence Rate Difference"),
                   aes(x = Subgroup, y = Estimate, color = model)) +
  geom_point(size = 2, position = position_dodge(width = .5)) +
  geom_errorbar(aes(ymin = `2.5% CL`, ymax = `97.5% CL`),
                width = 0.2,
                position = position_dodge(width = .5)) +
  scale_color_manual(values = c("#481567FF", "#3CBB75FF")) +
  theme_bw() +
  labs(x = "",
       y = str_wrap('Incidence rate difference of
                    cardiovascular disease or death
                    (cases/100 person-years)', width = 32),
       color = "") +
  geom_hline(aes(yintercept = hline),
             color = "red",
             linetype = "dashed",
             alpha = 0.3) +
  theme(legend.position = "none")

rate.ratio = ggplot(df.combined %>%
                      filter(Parameter == "Incidence Rate Ratio"),
                   aes(x = Subgroup, y = Estimate, color = model)) +
  geom_point(size = 2, position = position_dodge(width = .5)) +
  geom_errorbar(aes(ymin = `2.5% CL`, ymax = `97.5% CL`),
                width = 0.2,
                position = position_dodge(width = .5)) +
  scale_y_continuous(trans = "log2") +
  scale_color_manual(values = c("#481567FF", "#3CBB75FF")) +
  theme_bw() +
  labs(x = "",
       y = str_wrap('Incidence rate ratio of
                    cardiovascular disease or death
                    (shown on natural log scale)', width = 32),
       color = "") +
  geom_hline(aes(yintercept = hline),
             color = "red",
             linetype = "dashed",
             alpha = 0.3) +
  theme(legend.position = "bottom")

ggarrange(rate.ratio, rate.diff, ncol = 2, common.legend = T, legend = "bottom",
          labels = c("A", "B"), widths = c(1, 1))
```

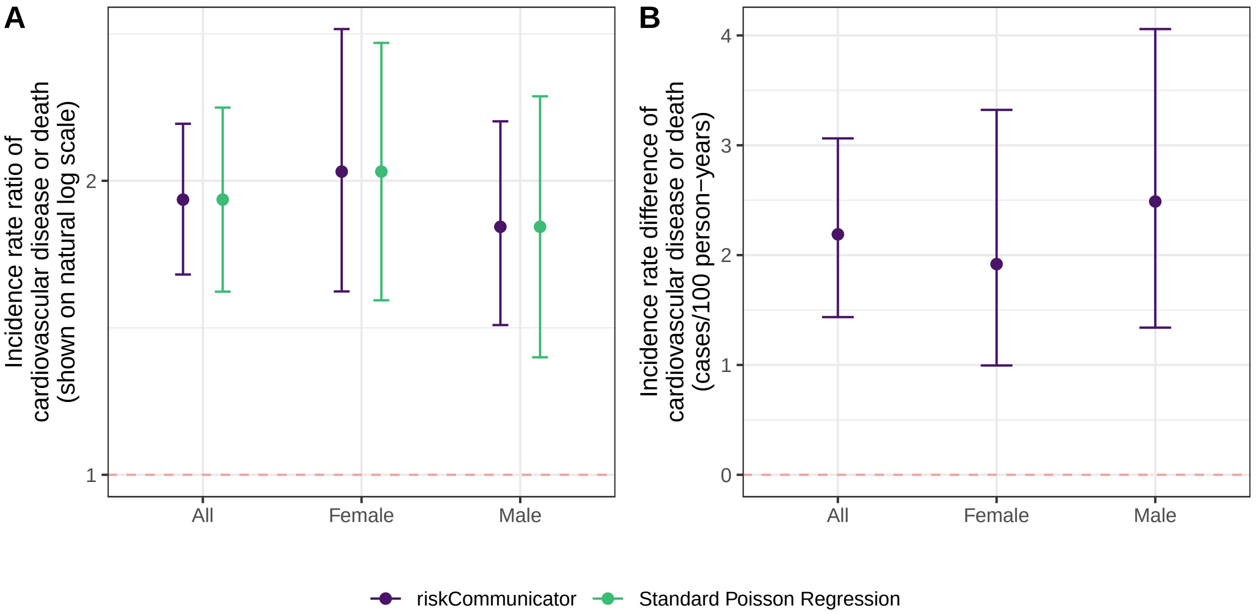


```
sessionInfo()
#> R version 4.2.0 (2022-04-22)
#> Platform: x86_64-apple-darwin17.0 (64-bit)
#> Running under: macOS Catalina 10.15.7
#>
#> Matrix products: default
#> BLAS:   /Library/Frameworks/R.framework/Versions/4.2/Resources/lib/libRblas.0.dylib
#> LAPACK: /Library/Frameworks/R.framework/Versions/4.2/Resources/lib/libRlapack.dylib
#>
#> locale:
#> [1] en_US.UTF-8/en_US.UTF-8/en_US.UTF-8/C/en_US.UTF-8/en_US.UTF-8
#>
#> attached base packages:
#> [1] stats     graphics  grDevices utils     datasets
#> [6] methods   base
#>
#> other attached packages:
#>  [1] ggpubr_0.4.0            sandwich_3.0-1
#>  [3] formatR_1.12            printr_0.2
#>  [5] forcats_0.5.1           stringr_1.4.0
#>  [7] dplyr_1.0.9             purrr_0.3.4
#>  [9] readr_2.1.2             tidyr_1.2.0
#> [11] tibble_3.1.7            ggplot2_3.3.6
#> [13] tidyverse_1.3.1         riskCommunicator_1.0.0
#>
#> loaded via a namespace (and not attached):
#>  [1] lattice_0.20-45  lubridate_1.8.0  zoo_1.8-10
#>  [4] assertthat_0.2.1 digest_0.6.29    utf8_1.2.2
#>  [7] R6_2.5.1         cellranger_1.1.0 backports_1.4.1
#> [10] reprex_2.0.1     evaluate_0.15    highr_0.9
#> [13] httr_1.4.3       pillar_1.7.0     rlang_1.0.2
#> [16] readxl_1.4.0     rstudioapi_0.13  car_3.0-13
#> [19] rmarkdown_2.14   labeling_0.4.2   munsell_0.5.0
#> [22] broom_0.8.0      compiler_4.2.0   modelr_0.1.8
```

```
#> [25] xfun_0.31        pkgconfig_2.0.3  htmltools_0.5.2
#> [28] tidyselect_1.1.2 gridExtra_2.3    codetools_0.2-18
#> [31] fansi_1.0.3      crayon_1.5.1     tzdb_0.3.0
#> [34] dbplyr_2.1.1     withr_2.5.0      MASS_7.3-57
#> [37] grid_4.2.0       jsonlite_1.8.0   gtable_0.3.0
#> [40] lifecycle_1.0.1  DBI_1.1.2        magrittr_2.0.3
#> [43] scales_1.2.0     cli_3.3.0        stringi_1.7.6
#> [46] carData_3.0-5    farver_2.1.0     ggsignif_0.6.3
#> [49] fs_1.5.2         xml2_1.3.3       ellipsis_0.3.2
#> [52] generics_0.1.2   vctrs_0.4.1      cowplot_1.1.1
#> [55] boot_1.3-28      tools_4.2.0      glue_1.6.2
#> [58] hms_1.1.1        abind_1.4-5      fastmap_1.1.0
#> [61] yaml_2.3.5       colorspace_2.0-3 rstatix_0.7.0
#> [64] rvest_1.0.2      knitr_1.39       haven_2.5.0
```
